# Prognostic impact of mitochondrial DNA D-loop variations in pediatric acute myeloid leukemia

**DOI:** 10.18632/oncotarget.26665

**Published:** 2019-02-12

**Authors:** Anudishi Tyagi, Raja Pramanik, Sreenivas Vishnubhatla, Radhika Bakhshi, Sameer Bakhshi

**Affiliations:** ^1^ Department of Medical Oncology, Dr. B. R. A. Institute Rotary Cancer Hospital, All India Institute of Medical Sciences, New Delhi, India; ^2^ Department of Biostatistics, All India Institute of Medical Sciences, New Delhi, India; ^3^ Department of Biomedical Sciences, Shaheed Rajguru College of Applied Sciences, University of Delhi, New Delhi, India

**Keywords:** pediatric AML, mitochondrial DNA, D-loop, prognosis, leukemia

## Abstract

The role of mitochondrial DNA (mt-DNA) changes, especially those in the regulatory D-loop region in Acute Myeloid Leukemia (AML) remains investigational. Consecutive 151 *de novo* pediatric AML patients, (≤18 yr) were prospectively enrolled from June 2013-August 2016, to assess the prognostic impact of mt-DNA D-loop variations (somatic/germline) on survival. For each patient, D-loop region was sequenced on baseline bone marrow and buccal swab, and mother’s blood sample. In 151 AML subjects, 1490 variations were found at 237 positions; 80.9% were germline and 19.1% somatic. The mean number of variations per position was 6.3. Variations with frequency ≥6 were analyzed for their impact on survival and 4 categories were created, namely “somatic-protective”, “somatic-hazardous”, “germline-protective” and “germline- hazardous”. Although, somatic-protective could not predict event free survival (EFS) or overall survival (OS), somatic-hazardous [(OS) HR = 2.33, *p* = 0.06] and germline-hazardous [(OS) HR = 2.85, *p* < 0.01] significantly predicted OS and EFS. Notably, the germline-protective, could significantly predict EFS (HR = 0.31, *p* = 0.03) and OS (HR = 0.19, *p* < 0.01), only when variations at ≥2 positions were present. On multivariate analysis, three positions namely 16111, 16126, 16362 and karyotype were found to be predictive of EFS. A prognostic index (PI) was developed using nomogram PI = (0.8*karyotype) + (1.0*c16111) + (0.7*t16362) + (1.2*t16126). Hazard ratio for EFS increased significantly with increasing PI reaching to a maximum of 3.3 (*p* < 0.01). In conclusion, the impact of mt-DNA D-loop variations on outcomes in pediatric AML depends on their nature (germline/somatic), position and mutational burden, highlighting their potential role as evolving prognostic biomarkers.

## INTRODUCTION

Mitochondria are a unique organelle and so is the mitochondrial DNA (mt-DNA). The human mitochondrial genome is 16,569 base pair (bp) in length, with double-stranded circular DNA molecules containing 37 genes [1]. A High turnover, lack of histones protection and poor proof reading ability of mt-DNA polymerase gamma render it highly susceptible to damage. Further, its close proximity to the electron transport chain, which generates reactive oxygen species (ROS), makes it even more prone to damage [2]. Lack of introns in mt-DNA ensures that most mutations occur in the coding sequence leading to biological consequences. Role of mitochondria in various human malignancies including leukemia has been long proposed and explored with varying outcomes [3–5]. This genome also includes a non-coding displacement region (D-loop) which consists of 1122 bp (16024 – 577 bp) of mitochondrial DNA. It acts as a promoter region for both the heavy and light strands of the mt-DNA, and contains essential transcription and replication elements [6]. It contains three hyper variable regions (HV1 at positions 16024–16383, HV2 at positions 57–372 and HV3 at positions 438–574). The D-loop is a hot spot region for mt-DNA variations. Genetic variability in the D-loop region has been suggested to affect the function of the respiration chain, leading to high ROS levels and instability in the mt-DNA [7, 8]. Thus, it is not surprising that mitochondrial dysfunction has been linked to human degenerative diseases and cancers, including leukemia [9, 10].

AML is a heterogeneous disease characterized by different cytogenetic aberrations, acquired mutations and impaired gene expression. Zhou *et al.*, 2017 recently reported that polymorphism T152C in the Dloop region was associated with particularly AML-M3 subtype [11]. A previous study from our group reported that some of the mitochondrial D-loop variations were significantly associated with inferior survival in pediatric AML [12].

Our group recently published a descriptive analysis of mt-DNA D-loop variations among Indian children with AML [13]. However, the prognostic impact of somatic and germline mt-DNA D-loop variations in large cohort of AML subjects have never been reported. In view of these lacunae, the present study was conducted with the primary objective of assessing the impact of mitochondrial D-loop variations (somatic/germline) on outcomes in pediatric AML.

## RESULTS

A total of 200 patients were registered at the centre during the study period. Out of 200 subjects, 49 patients were excluded from the study, (5 patients were acute promyelocytic leukemia, 15 patients had only one visit, 4 patients had an unsatisfactory buccal swab, 15 patients had failed sequencing and for 10 patients’s the mother sample was not available). Therefore, 151 patients were eligible for the study.

In this sample of 151 eligible patients, median age was 10 years (0.7 to 18 years); male: female ratio was 2.5:1. Cytogenetics was evaluable in 125 patients (82.8%); 48.8% of the patients had good risk cytogenetics while intermediate and poor risk cytogenetics was present in 39.2% and 12.0% subjects respectively (Supplementary Table 1).

CR with induction therapy was achieved by 123 (81.5%) patients and 85 patients subsequently relapsed/died after achieving remission. Median follow up was 33.8 months. The 2-year EFS (±SE) and OS was 32.3 ± 3.9% and 44.1 ± 4.2% respectively.

### Relationship of baseline patients characteristics with EFS and OS

In univariate analysis, adverse cytogenetics risk group was significantly associated with inferior outcome whereas presence of chloromas was found to be significantly associated with better outcome (Table 1).

**Table 1:** Association of baseline patients’ characteristics with survival outcome (months)

Parameters (*n =* 151)	Event free survival			Overall Survival		
	Median	HR, (95%CI)	*P*	Median	HR, (95%CI)	*P*
Age (Years) ≤10 (*n* = 77) ≥10 (*n* = 74)	9.4 12.2	1.00 0.8 (0.6–1.3)	0.42	18.9 24.1	1.00 1.6 (0.9–2.7)	0.06
Sex Male (*n* = 108) Female (*n* = 43)	11.2 9.4	1.00 1.3 (0.8–1.9)	0.29	20.1 14.2	1.00 1.2 (0.7–1.8)	0.55
Hemoglobin (g/dl) ≤8 (*n* = 82) ≥8 (*n* = 69)	8.4 12.7	1.00 1.4 (0.9–2.1)	0.09	13.8 21.7	1.00 1.0 (0.6–1.7)	0.95
TLC (/mm^3^) ≤11,000 (*n* = 55) ≥11,000 (*n* = 96)	11.4 9.7	1.00 1.2 (0.7–1.8)	0.43	21.3 16.3	1.00 1.1 (0.7–1.8)	0.57
Platelets (/mm^3^) ≤50,000 (*n* = 48) ≥50,000 (*n* = 103)	10.9 10.2	1.00 1.1 (0.7–1.7)	0.65	21.3 19.9	1.00 1.0 (0.7–1.7)	0.84
CSF (*n =* 105) Negative (*n* = 92) Positive (*n* = 13)	12.1 9.3	1.00 1.5 (0.8–2.9)	0.18	21.7 18.9	1.00 1.3 (0.6–2.7)	0.44
Chloromas Negative (*n* = 126) Positive (*n* = 25)	9.4 24.0	1.00 0.4 (0.2–0.8)	0.01	13.3 24.0	1.00 0.4 (0.2–0.8)	0.01
Cytogenetic risk (*n =* 125) Favorable (*n* = 61) Intermediate (*n* = 49) Adverse (*n* = 15)	12.7 11.2 5.9	1.00 1.2 (0.7–1.9) 2.2 (1.2–4.3)	0.02	24.0 20.3 8.6	1.00 1.3 (0.8–2.3) 3.1 (1.6–6.2)	0.002
FLT3-ITD (*n =* 127) Negative (*n* = 117) Positive (*n* = 10)	10.9 5.5	1.00 1.4 (0.6–3.0)	0.39	18.9 6.4	1.00 1.8 (0.8–3.9)	0.16
NPM1 (*n =* 122) Negative (*n* = 113) Positive (*n* = 9)	10.9 8.2	1.00 1.4 (0.6–3.0)	0.41	20.1 11.7	1.00 1.4 (0.6–3.3)	0.43
^*^AML-ETO (*n =* 125) Negative (*n* = 76) Positive (*n* = 49)	10.9 11.4	1.00 0.9 (0.6–1.4)	0.68	20.1 21.8	1.00 0.8 (0.5–1.4)	0.48

Abbreviations: CI, Confidence interval; HR, Hazard ratio; TLC, Total leukocyte count; CSF, Cerebrospinal fluid; FLT3-ITD, *FMS* like tyrosine kinase-3 internal tandem duplication; NPM1, Nucleophosmin1; AML-ETO, Acute myeloid leukemia eight twenty one.

^*^Done by cytogenetics.

### Mitochondrial D-Loop variations

All patients had one or more mitochondrial D-loop variations. A total of 1490 variations were identified at 237 positions in the D-Loop; 855 positions did not have variations. Thus, the mean number of variations per mutated position for the entire D-loop region was 6.3. Of these 1490 variations; 1206 (80.9%) variations were germline and 284 (19.1%) variations were somatic. One hundred and four, out of 237 positions in mtDNA had not been previously reported when compared with available databases updated to 17.12.17. All the variations have been submitted to the bankit (NCBI) at https://www.ncbi.nlm.nih.gov/WebSub/?tool=genbank vide accession number; GenBank “MG816363-MG816455”.

### Relationship of D-loop variations with EFS

Of the 237 positions with variations, 40 positions were affected with variations in ≥6 patients (mean frequency of variation in the D-loop region was 6.3). Univariate analysis revealed that among somatic variations, 8 positions (16111, 16126, 16189, 16209, 16278, 16304, 151 and 204) were found to be significantly associated with inferior EFS. Among germline variations, position 16126 was significantly associated with inferior EFS. On combining both somatic and germline variations (any variations), three positions (16111, 16126 and 482) were significantly associated with inferior EFS (Supplementary Table 2 and Supplementary Table 3).

D-loop variations, thus identified, were categorized into four different groups namely somatic protective, somatic hazardous, germline protective, germline hazardous (Table 2). Somatic protective group did not have any association with EFS; whereas the other three categories predicted EFS. Notably, in germline protective group the association was significantly seen when variations at ≥2 positions were observed (Table 2 and Supplementary Figure 1).

**Table 2:** Event free survival of patients with variations into four categories of prognostic groups

Type of variations (prognostic groups)	Specific positions of significance	*N*	Median Survival Months	HR	*P*
Somatic Protective Wild Variations	16184, 150, 198, 489	57 10	10.9 19.3	1.00 0.65	-- 0.36
Somatic Hazardous Wild Variations	16051, 16093, 16111, 16126, 16189, 16209, 16278, 16304, 16311, 16362, 16390, 151, 152, 204, 482	26 12	15.4 8.2	1.00 2.46	-- 0.02
Germline Protective Wild Variation at 1 position Variation at ≥2 positions	16278, 16318, 73	6 118 18	3.9 10.3 15.4	1.00 0.59 0.31	-- 0.25 0.03
Germline Hazardous Wild Variations	16126, 16172, 16327, 16390, 482	113 29	12.7 5.9	1.00 2.27	-- ≤0.01

Abbreviations: HR, Hazard ratio; N, Number of patients.

In each prognostic groups, EFS of patients with variations have been compared to wild type.

### Relationship of D-loop variations with OS

Univariate analysis revealed that somatic variations at 7 positions viz. 16111, 16126, 16209, 16278, 16304, 151 and 204 had significantly inferior OS while germline variations at two sites, namely 16126 and 146, were significantly associated with inferior OS. When somatic and germline variations were combined (any variation), four positions viz 16111, 16126, 146 and 482 were significantly associated with inferior OS whereas variations at position 16318 was significantly associated with better OS (Supplementary Table 4 and Supplementary Table 5).

**Figure 1:**
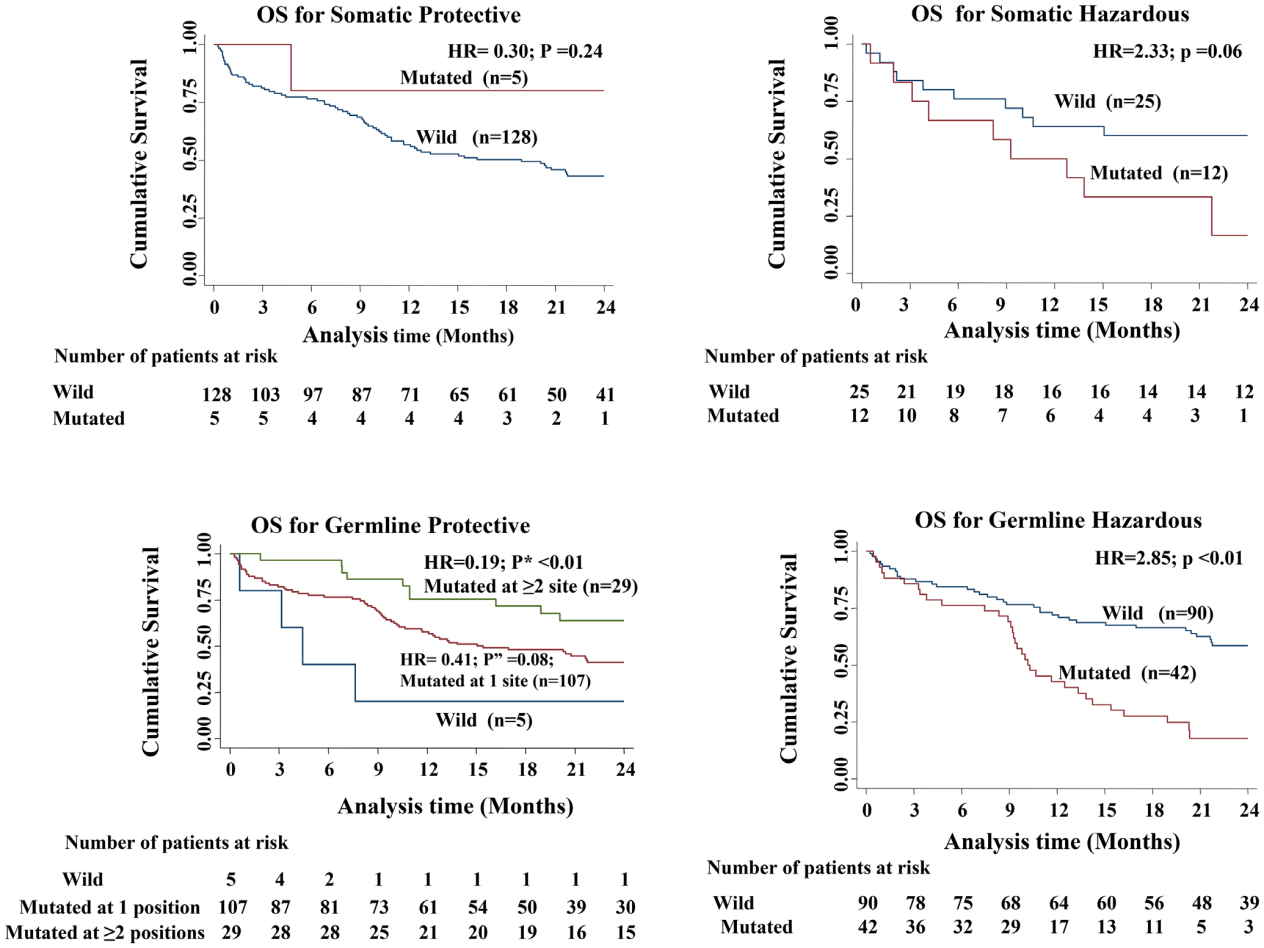
Kaplan–Meier curves comparing the overall survival among the four different categories of variations (*p* value shows the log rank of comparison between the survival curves of wild type patients versus those with variation and *p*” value shows the log rank of comparison between the survival curves of wild type patients versus those with variation at 1 position; P^*^ value shows the log rank of comparison between the survival curves of wild type patients versus those with variation at 2 positions)

D-loop variations, thus identified, were categorized into four different groups namely somatic protective, somatic hazardous, germline protective, germline hazardous (Table 3). Somatic protective group did not have any association with OS; whereas the other three categories predicted OS. Notably, in germline protective group the association was significantly seen when variation at ≥2 positions were observed (Table 3 and Figure 1).

**Table 3:** Overall survival of patients with variations into four categories of prognostic groups

Type of variations (prognostic groups)	Specific positions of significance	*N*	Median Survival Months	HR	*P*
Somatic Protective Wild Variations	16184, 150, 198	128 5	16.2 --	1.00 0.30	-- 0.24
Somatic Hazardous Wild Variations	16051, 16093, 16111, 16126, 16192, 16209, 16278, 16304, 16311, 16327, 16362, 16390, 151, 152, 204, 482	25 12	-- 9.3	1.00 2.33	-- 0.06
Germline Protective Wild Variation at 1 position Variation at ≥2 positions	16209, 16278, 16304, 16318, 73	5 107 29	3.1 15.1 --	1.00 0.41 0.19	-- 0.08 ≤0.01
Germline Hazardous Wild Variations	16126, 16172, 16327, 16390, 482	90 42	-- 10.2	1.00 2.85	-- ≤0.01

Abbreviations: HR, Hazard ratio; N, Number of patients.

In each prognostic groups: OS of patients with variations have been compared to wild type.

### Multivariate analysis for outcome

On multivariate analysis, karyotype and any variation at positions 16111, 16126 and 16362 emerged as independent prognostic factors for poor EFS whereas in OS, karyotype along with any variations at positions 16111 and 16126 emerged as independent prognostic factors (Table 4).

**Table 4:** Factors associated with survival based on multivariate analysis

Variables in model	Event free survival			Overall survival		
	Hazard coefficient	HR (95%CI)	*P*	Hazard coefficient	HR (95%CI)	*P*
Cytogenetic risk (^*^others Vs adverse)	0.8	2.2 (1.2–4.2)	0.01	1.1	2.9 (1.5–5.7)	0.001
16111	1.0	2.8 (1.2–6.5)	0.02	1.4	4.1 (1.6–9.8)	0.002
16126	1.2	3.2 (1.6–6.7)	0.002	0.9	2.6 (1.2–5.7)	0.01
16362	0.7	1.9 (1.1–3.6)	0.02	0.4	1.6 (0.8–3.2)	0.18

Abbreviations: CI, Confidence interval; HR, Hazard ratio.

*Others - Favourable and intermediate ELN risk group.

### Development of the prognostic model

The hazard coefficient for each of the above variables independently predictive of EFS was calculated. On the basis of these hazard coefficients, the prognostic index (PI) for each individual patient was calculated using the following formula: PI = (0.8*karyotype) + (1.0*c16111) + (1.2*t16126). (0.7*t16362) (Table 4).

**Figure 2:**
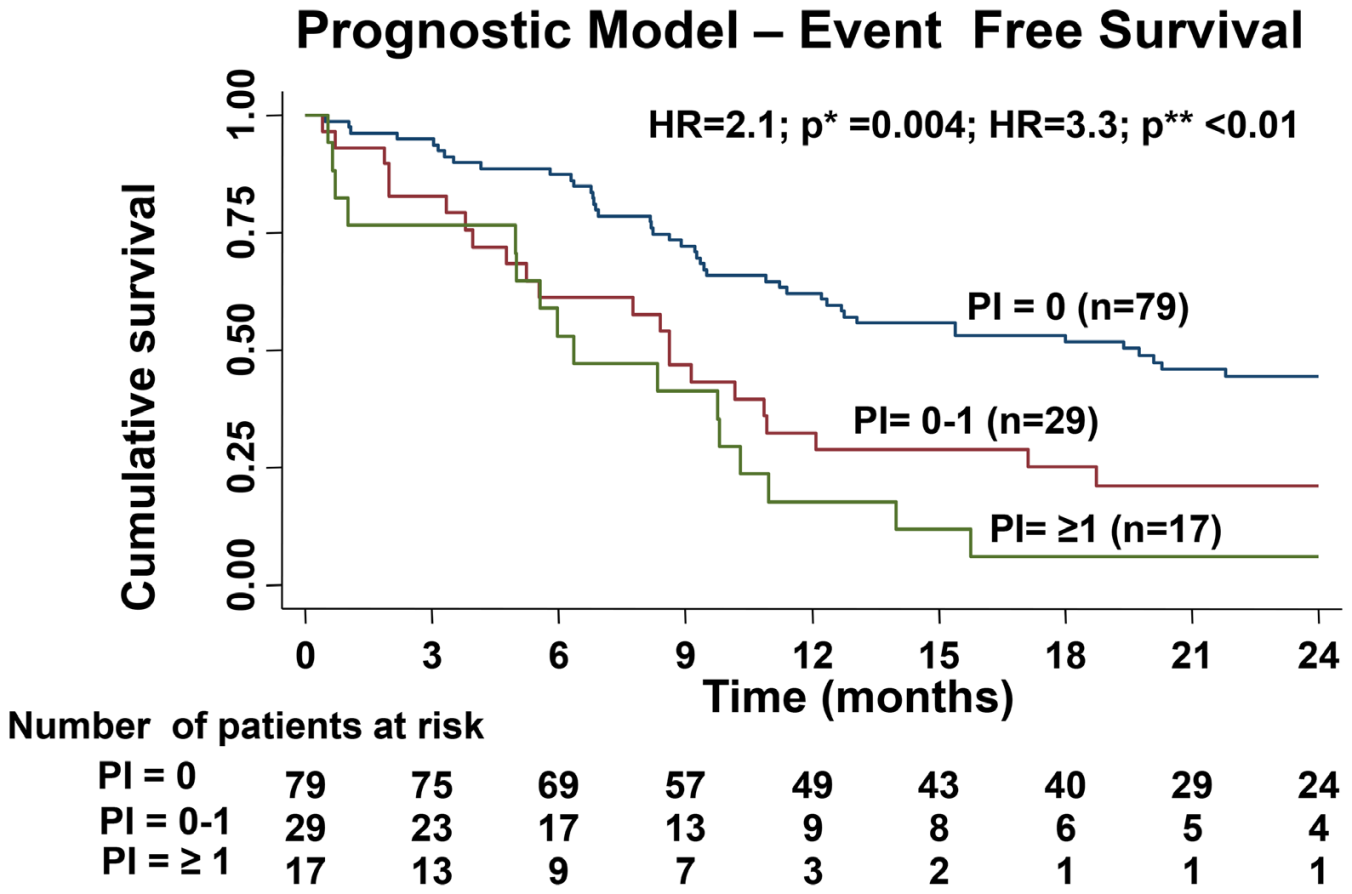
Difference in EFS of the three cohorts based on the prognostic index created by hazard coefficient of all the significant predictors for EFS in multivariable analysis (p^*^ value shows the comparison the survival curves prognostic index (PI) PI = 0 versus PI = 0–1 and p^**^ value shows the comparison between the survival curves of PI = 0 versus PI ≥ 1)

Based on this, median EFS of 3 different PI categories (PI = 0, PI ≥ 0 to ≤1 and PI ≥ 1) is shown in Figure 2. The median survival curves separate significantly (p^*^ = 0.004 and p^**^ < 0.01) with better EFS for patients with PI score ≤0 and the worst EFS for those with the PI score ≥ 1 (Figure 2). The model could not accurately predict the OS.

### Impact of change in nucleotide

We analyzed our data in term of the quantum of change from one type of nucleotide to the other both in the somatic and germline categories separately and its impact on survival. Any somatic variation that involves a change into T from other nucleotide was noted in 64 patients and was associated with inferior OS (*p* = 0.02) (Supplementary Table 6). On analyzing the quantitative burden of such changes, we observed that a higher burden (>2 positions) of such variations was significantly associated with inferior overall survival (*p* = 0.02) (Supplementary Table 6).

## DISCUSSION

Variations in the mitochondrial DNA D-loop region have been observed in different solid malignancies and their incidence varies from 21% to 80% depending on the cancer type [13, 14–21]. Although, several studies have reported alterations in mt-DNA in hematological malignancies, studies elucidating the role of D-loop variations in AML and their prognostic significance are only a few [11, 12]. Studies focusing exclusively on AML suggest that these variations may play a role in the development, response to treatment and prognosis of AML [11, 12, 22].

The previous study from our group showed for the first time the impact of D-loop variations on EFS on pediatric AML; in that study, we identified three variations (16126, 16224 and 16311) in the hyper variable (HV1) region that predicted inferior EFS [12]. In the present study we went further ahead, classified the variations from a different set of 151 new subjects according to their germline and somatic status, and tried to identify unique prognostic groups.

In the present study, we could identify variations in 100% of patients as compared to 79% (*n* = 44) by Surender K. Sharawat et al., 2010 [12] and 60% (*n* = 18) by Yao et al., 2007 [5]. Our data, however, concurs with Zhou et al., 2017 [11] and Silkjaer et al., 2013 [22] who also reported variations in 100% of their AML subjects. Interestingly, Zhou et al., 2017 had sequenced only the D-loop region while Silkjaer et al., 2013 sequenced the whole mtDNA [11, 22]. Strikingly, none of these studies separately identified these variations as germline or somatic. Thus, the classification of variations as germline or somatic constitutes a novel information in this field.

We observed that variations at certain positions, depending on their germline status, predicted a better or worse survival. It was even more interesting to note that the effect of germline protective variations was dependent on the number of variation positions in the individual. It appears as if both the characteristics and the burden of mt-DNA variations influence the phenotype of the disease and ultimately its biological behavior and survival outcomes. In the current era of precision medicine, tumor mutational burden (TMB) is being used as biomarker predicting response to immune-therapies in solid malignancies. In the recent phase-3 trial, immunotherapy was found to be significantly better than chemotherapy in the frontline treatment of metastatic non small cell lung cancer (NSCLC) with high TMB (>10 mutation per mega base) [23]. In this context, our observation that the burden of mt-DNA variations influences outcomes in AML appears highly significant and thought provoking.

We developed a prognostic model using the variations at three positions viz. 16111, 16126, 16362 and karyotype as prognostic variables and could demonstrate significant survival differences between patients with different prognostic indices. Although this predictive model needs to be validated in a larger cohort, nevertheless it is an important first attempt to integrate the novel biomarker in the prognostication of AML. Although, variations at 16111 and 16362 have not been reported to affect AML pathogenesis and prognosis in literature previously, their role has been reported in prostate cancer risk [24].

T16311C was reported to predict an inferior EFS by Surender K. Sharawat et al., 2010 [11] and OS (univariate analysis only) by Silkjaer et al., 2013 [22]. However, it was later found to be significantly associated with the favorable prognostic group comprising of APML, inv16 or t (8; 21) [22]. In our study, we could not demonstrate any significant association of these variations either with EFS or OS.

One of the limitations of our study is that we sequenced only D-loop region of the mitochondria in our subjects; studies also need to be done on the whole mitochondrial genome as other part of the mt-DNA may also have a role in its pathogenesis. In term of methodology, we used PCR for amplification prior to sequencing which has its own inherent error. In order to increase the specificity of the reaction, we performed nested PCR and PCR products were sequenced in both forward and reverse direction. Further the prognostic model, which we developed using the variations at positions (16111, 16126 and 16362), needs to be validated in a larger independent cohort.

The strength of our study lies in the uniformity of the treatment protocol used and use of corresponding DNA from mothers to identify germline and somatic variations. Instead of bluntly separating out patients with or without variations, we categorized variations into four prognostically relevant groups namely somatic protective, somatic hazardous, germline protective and germline hazardous. The present paper is the first to report a prognostic model in AML integrating mt-DNA D-loop variations as prognostic biomarkers. This may be a useful tool to refine the prognostic stratification of AML, an area of unmet need.

## MATERIALS AND METHODS

### Patient’s sample

This was a single centre, prospective cohort study conducted on *de novo* pediatric (≤18 year) patients with AML at the tertiary cancer centre at our institute, who were registered between June 2013 to August 2016. Study was approved by the institute ethics committee. Informed consent was taken from guardian of patients and assent form from those subject’s ≤8 years of age. We excluded patients with acute promyelocytic leukemia (APML) and myelodysplastic syndromes (MDS) related AML. We sampled bone marrow (BM) and buccal swab from each patient and collected blood samples from the mother’s of the patients. Patients whose mother’s sample was not available were excluded from the study.

### DNA extraction

Total DNA was isolated from 200 µl of patient’s BM sample and patient’s mother sample using the QI-Aamp DNA Mini-Kit (Qiagen, Hilden, Germany). DNA from buccal swab of the patients was isolated using G-biosciences DNA extraction kit. The quantity and quality of the DNA was assessed by spectrophotometer and agarose gel electrophoresis respectively.

### Nested polymerase chain reaction (PCR)

The D-loop region was amplified by nested PCR to increase the specificity. All the primers were high performance liquid chromatography (HPLC) purified and details of the primers sequences are given below.

**Table:** 

Primer	Sequence
Outer Primer Forward	5′-TCCACCATTAGCACCCAAAG-3′
Outer Primer Reverse	5′-GGGGATGCTTGCATGTGTA-3′
D1F	5′-TCCACCATTAGCACCCAAAG-3′
D1R	5′-GCTGTGCAGACATTCAATTGTT-3′
D2F	5′-GAGCTCTCCATGCATTTGGT-3′
D2R	5′-GGGGATGCTTGCATGTGTA-3′

PCR conditions for amplification for the whole D-loop region were: initial denaturation at 94°C for 3 min; 35 cycles of denaturation at 94°C for 45 s; annealing at 58°C for 1 min; elongation at 74°C for 30 s The PCR master mix contained 1.25 mmol/l of each dNTP (Fermentas, Glen Burnie, MD, USA), 20 pmol of each primer, 10 mmol/l Tris–HCl (pH 9), 50 mmol/l KCl, 1.5 mmol/l MgCl2 and Taq DNA polymerase (5 u/µl) (New England Biolabs, Ipswich, MA) in a total volume of 50 µl. PCR conditions for internal fragments of the D-loop were as follows: initial denaturation at 94°C for 3 min; 35 cycles of denaturation at 94°C for 30 s; annealing at 56°C for 30 s; elongation at 72°C for 45 s with a final extension at 72°C for 8 min (Verti, Applied Biosystem; USA). PCR products were checked by resolving on a 2.0% agarose gel.

### Nucleotide base sequencing and identification of variations

PCR product was eluted and purified from the gel using a column based PCR purification kit (Qiagen, Hilden, Germany). The purified product was quantified and sequenced using Sanger’s sequencing (ABI 3730 XL; Applied Biosystems Instruments, Foster City, CA, USA). Obtained sequences and chromatograms were examined using DNA star software (Laser gene 14.1; USA) with reference sequence (NC_012920.1). Furthermore, all sequences were compared with Cambridge mitomap database (http://www.mitomap.org/bin/view/MITOMAP; updated on 17 Jan 2018) for already known polymorphisms and mutations. The available databases also include the polymorphisms and mutations observed in healthy individual in India that overlaps with those observed in other countries; however, there is no separate database for mitochondrial D-loop polymorphisms in Indian subjects [25, 26]. Therefore, we preferred to designate the D-loop sequence alterations from the database in our subjects as variations instead of mutations or polymorphisms.

### Baseline cytogenetics and molecular analysis

Pretreatment samples from all patients underwent cytogenetics analysis at NABL accredited laboratory and chromosomal abnormalities were described according to the International System for Human Cytogenetic Nomenclature (ISCN) [27]. Molecular analysis for FLT3-ITD and NPM1 were done using PCR [28, 29]. Based on this information, the European Leukemia Network (ELN) classification was used to categorize the patients into three different prognostic risk groups; good, intermediate and adverse risk [30].

### Treatment

All patients were treated on a uniform protocol. They were induced with 3+7 regimen (daunorubicin 60 mg/m^2^ for 3 days and cytosine arabinoside 100 mg/m^2^ as a 24 hour continuous infusion for 7 days). Patients, who did not achieve complete remission (CR) after 1st induction, were given ADE (Cytarabine; 100 mg/m^2^ BID day1–10 Daunorubicin; 50 mg/m^2^ day1–3 and Etoposide; 100 mg/m^2^ day1–3) as a 2nd induction [31]. CR was defined as bone marrow blast <5%, absolute neutrophil count >1000/uL, platelet count >100000/uL, no residual evidence of extramedullary disease and the child being independent of transfusion [32]. After achieving complete remission (CR), the patients received three cycles of high dose cytosine arabinoside at 18 g/m^2^. Twenty eight patients received consolidation at 12g/m^2^ as a part of a randomized controlled trial. Relapse following CR was defined as reappearance of leukemic blast in peripheral blood or the finding of >5% blasts in the bone marrow, not attributable to another cause [32]. Salvage chemotherapy was attempted for relapse and patients received allogeneic transplantation in CR2, if a matched sibling was available.

### Sample size calculation

Our earlier work indicated that subjects with atleast 3 variations had higher mortality (HR = 2.03) although, statistically not significant (*p* = 0.20) [11]. To declare this excess of hazards of mortality significant in a 2-sided log rank test with 80% power and 5% alpha error, we required 45 subjects with atleast 3 D-loop variations and 45 cases with <3 variations. During the course of the study we realized that minimum variations per patient in our study were 4. Further, all variations did not behave similarly (some protective versus some hazardous), which prompted us to arbitrarily add 60 more patients.

### Statistical analysis

Baseline characteristics for all the patients were summarized using median and range. Primary end point was overall survival (OS). OS was measured as the duration from the date of enrollment to death from any cause. Event free survival (EFS) was measured as the duration from date of enrollment to date of relapse or death due to disease. The data was censored at the date (22-December-2017) on last follow up for alive patients. The Kaplan–Meier statistics were used to estimate OS and EFS and log-rank test was used to compare differences between survival curves. *P* value ≤ 0.05 was considered significant. All statistical analysis was done using Stata 11.2.

### Classification and categorization of variations

Germline variations were defined as those which were detected in all the three samples (patient’s BM, buccal swab and mother’s blood) whereas somatic variations were those which were present in patient’s BM sample. Further to check the impact of these variations on EFS and OS, whether these variations are protective or hazardous, we performed the Cox proportional hazard analysis. We selected only those positions where variations were present in ≥6 patients as the mean frequency of variation in the D-loop was 6.3. We got 40 such positions and calculated the hazard ratio for EFS for each individual positions using Cox proportional hazard model and restricted mean survival time (RMST). All positions with hazard ratio ≤0.8 and *p* value ≤0.25 were considered protective. All positions with hazard ratio ≥1.2 and *p* value ≤0.25 were considered hazardous. Those with hazard ratio between ≤0.8 and ≥1.2 were not considered for analysis. The above values were taken because we wanted to include all position that showed a trend towards significance. We wanted to be inclusive at this stage and therefore relaxed the criteria. We then examined the effect of these four categories on survival. This time we used the Cox proportional hazard model and kept a strict threshold of *p* value ≤0.05 for determining statistical significance. Similar analysis was done on the overall survival.

### Multivariate analysis

Variations in the D-Loop region of mt-DNA were compared with the wild type using Cox proportional hazards model for EFS and OS. All factors which were significant in the univariate analysis (*p* < 0.05) were considered for multivariate analysis. Stepwise multivariate Cox regression method was employed to evaluate the independent prognostic factors. Prognostic index (PI) of individual variations was calculated with hazard coefficient. Using this index, nomogram for predicting EFS was developed.

## SUPPLEMENTARY MATERIALS FIGURES AND TABLE


