# Supplementary material for: Prognostic impact of mitochondrial DNA D-loop variations in pediatric acute myeloid leukemia
Source: Oncotarget. 2019 Feb 12;10(13):1334–43. doi: 10.18632/oncotarget.26665 (PMC6407682; doi:10.18632/oncotarget.26665)
Supplement: Supplementary file 1 [file oncotarget-10-1334-s001.pdf]

Prognostic impact of mitochondrial DNA D-loop variations in pediatric acute myeloid leukemia

SUPPLEMENTARY MATERIALS

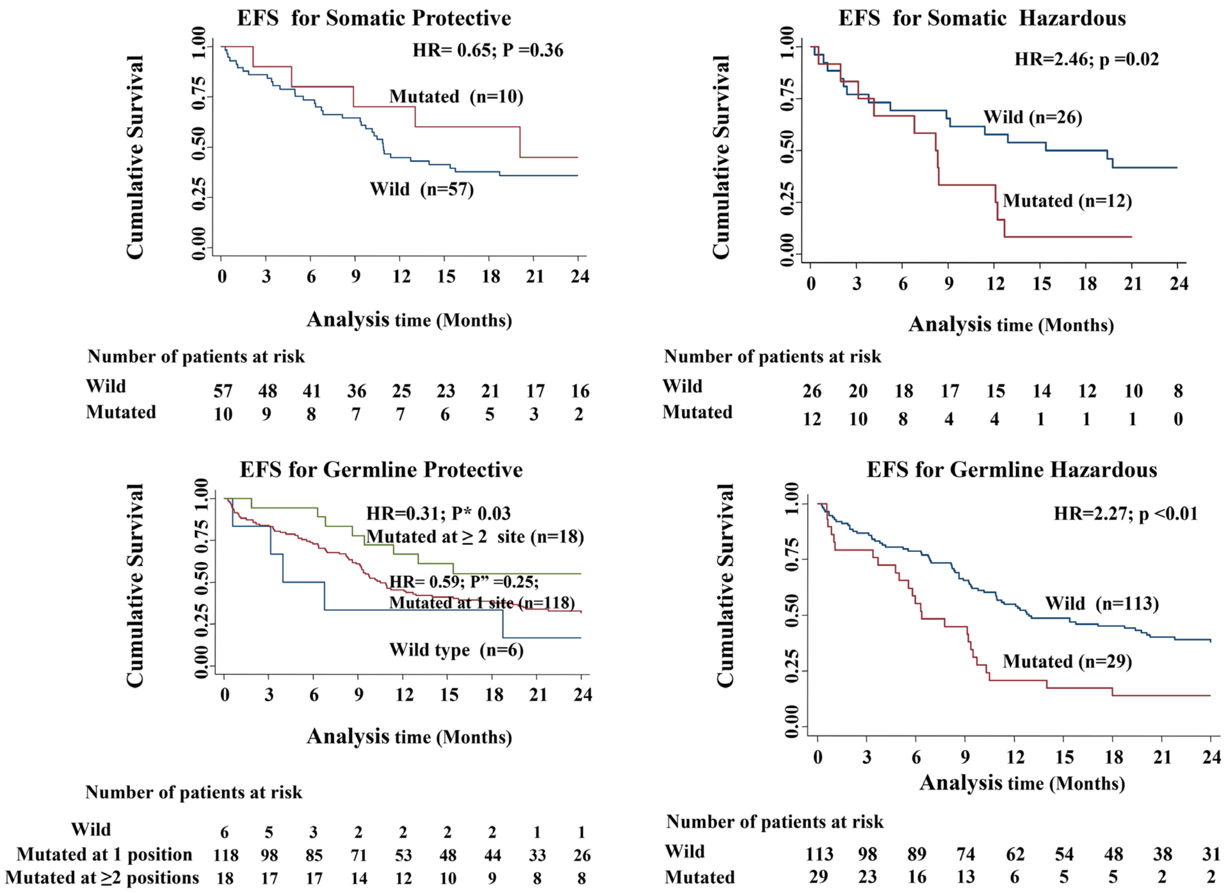

Supplementary Figure 1: Kaplan–Meier curves comparing the event free survival among the four different categories of variations. (*p* value shows the Log rank of comparison between the survival curves of wild type patients versus those with variation and *p*" value shows the Log rank of comparison between the survival curves of wild type patients versus those with variation at 1 position; P^* value shows the Log rank of comparison between the survival curves of wild type patients versus those with variation at ≥2 positions).

Supplementary Table 1: Baseline patient's characteristics

Parameters ($N = 151$)	Median (Range)	
Age	10 (0.7–18)	
Sex	M:F (2.5:1)	
Hemoglobin (g/dl)	7.8 (1.8–14.4)	
TLC (/mm^3)	17.1 (0.7–329.0)	
Platelets (/mm^3)	31.0 (1.0–583.0)	
Complete remission	123 (81.5%)	
Cytogenetic risk 125/151 (82.8%)	Good Risk	61 (48.8%)
	Intermediate Risk	49 (39.2%)
	Poor Risk	15 (12.0%)
*AML-ETO ($N = 125$)	49 (39.2%)	
NPM1 ($N = 122$)	9 (7.4%)	
FLT3-ITD ($N = 127$)	10 (7.9%)	

Abbreviations: *FLT3*-ITD, *FMS* like tyrosine kinase-3 internal tandem duplication; NPM1, Nucleophosmin; TLC, Total leukocyte count.

*Done by cytogenetics.

Supplementary Table 2: Event free survival for mitochondrial D-loop variations (Cox proportional)

Position and type of variations	*N*	Median Survival Months	Hazard ratio	*P*
16051				
Wild	140	10.8	1.00	--
Somatic	1	--	4.92	0.11
Germline	10	9.3	0.70	0.44
Any type of variations	11	9.3	0.81	0.63
16093				
Wild	143	10.9	1.00	--
Somatic	2	4.2	3.05	0.12
Germline	6	6.8	1.24	0.67
Any type of variations	8	6.8	1.54	0.30
16111				
Wild	144	10.9	1.00	--
Somatic	2	0.3	28.4	≤0.01
Germline	5	9.8	1.59	0.31
Any type of variations	7	1	2.18	0.05
16126				
Wild	135	12.2	1.00	--
Somatic	2	0.5	7.88	≤0.01
Germline	14	5.9	2.47	≤0.01
Any type of variations	16	5.6	2.70	≤0.01
16129				
Wild	131	10.9	1.00	--
Somatic	3	--	1.64	0.39
Germline	17	9.5	0.93	0.83
Any type of variations	20	9.5	1.03	0.93
16172				
Wild	142	10.9	1.00	--
Somatic	3	6.8	0.62	0.63
Germline	6	6.3	2.02	0.13
Any type of variations	9	6.8	1.46	0.37
16189				
Wild	142	10.9	1.00	--
Somatic	3	0.6	3.28	0.04
Germline	6	17.1	0.84	0.73
Any type of variations	9	8.4	1.23	0.59
16192				
Wild	144	10.8	1.00	--
Somatic	2	0.7	0.83	0.85
Germline	5	8.6	0.90	0.87
Any type of variations	7	8.6	0.88	0.81
16209				
Wild	143	10.3	1.00	--
Somatic	2	1.1	6.45	0.01
Germline	6	15.8	0.57	0.34
Any type of variations	8	10.5	0.90	0.82
16223				
Wild	14	10.9	1.00	--
Somatic	11	6.8	1.05	0.89
Germline	96	9.8	1.02	0.92
Any type of variations	107	9.7	1.02	0.91
16278				
Wild	138	10.5	1.00	--
Somatic	2	1.1	6.36	0.01
Germline	11	13.1	0.58	0.24
Any type of variations	13	6.8	0.78	0.54
16292				
Wild	145	10.5	1.00	--
Somatic	1	--	4.47	0.99
Germline	5	8.6	0.50	0.34
Any type of variations	6	8.6	0.41	0.21
16294				
Wild	145	10.8	1.00	--
Somatic	--	--	--	--
Germline	6	8.6	0.63	0.43
Any type of variations	6	8.6	0.63	0.43
16304				
Wild	144	10.5	--	--
Somatic	1	--	24.3	≤0.01
Germline	6	10.9	0.81	0.69
Any type of variations	7	9.4	1.01	0.98
16311				
Wild	120	10.3	1.00	--
Somatic	9	5.5	1.74	0.13
Germline	22	12.4	0.75	0.34
Any type of variations	31	11.2	0.96	0.87
16327				
Wild	144	10.9	1.00	--
Somatic	2	3.0	0.79	0.81
Germline	5	4.8	1.85	0.18
Any type of variations	7	4.8	1.51	0.32
16362				
Wild	129	10.9	1.00	--
Somatic	4	8.3	1.97	0.18
Germline	18	10.2	1.06	0.83
Any type of variations	22	10.2	1.20	0.49
16390				
Wild	145	10.9	1.00	--
Somatic	1	--	6.17	0.07
Germline	5	9.4	1.73	0.23
Any type of variations	6	9.4	1.96	0.11

16519				
Wild	27	9.7	1.00	--
Somatic	5	3.7	0.90	0.88
Germline	119	10.9	0.98	0.95
Any type of variations	124	10.9	0.98	0.94
61				
Wild	140	10.9	1.00	--
Somatic	11	10.2	1.28	0.48
Germline	0	--	--	--
Any type of variations	11	10.2	1.28	0.48
73				
Wild	6	3.9	1.00	--
Somatic	6	5.5	0.93	0.91
Germline	139	10.9	0.56	0.21
Any type of variations	145	10.9	0.57	0.22
146				
Wild	132	11.2	1.00	--
Somatic	6	5.6	1.29	0.62
Germline	13	8.9	1.47	0.25
Any type of variations	19	8.4	1.41	0.23
150				
Wild	130	10.3	1.00	--
Somatic	5	20.3	0.43	0.24
Germline	16	9.5	0.83	0.57
Any type of variations	21	12.2	0.72	0.28
151				
Wild	142	10.9	1.00	--
Somatic	1	--	14.6	0.01
Germline	8	9.4	1.13	0.76
Any type of variations	9	6.9	1.31	0.49
152				
Wild	101	11.4	1.00	--
Somatic	7	4.8	2.16	0.07
Germline	43	10.9	1.02	0.90
Any type of variations	50	9.4	1.13	0.56
195				
Wild	106	10.7	1.00	--
Somatic	9	6.8	1.10	0.81
Germline	36	12.4	0.82	0.42
Any type of variations	45	12.2	0.87	0.53
199				
Wild	145	10.9	1.69	0.99
Somatic	1	--	--	--
Germline	5	3.8	1.61	0.35
Any type of variations	6	8.3	1.11	0.84
204				
Wild	135	10.9	1.00	--
Somatic	2	0.53	31.1	≤0.01
Germline	14	10.3	0.72	0.40
Any type of variations	16	9.2	0.92	0.81
207				
Wild	142	10.5	1.00	--
Somatic	4	3.5	0.78	0.74
Germline	5	9.2	1.49	0.38
Any type of variations	9	9.2	1.19	0.66
263				
Wild	3	--	1.00	--
Somatic	9	10.9	0.29	0.09
Germline	139	10.5	0.44	0.16
Any type of variations	148	10.8	0.43	0.15
482				
Wild	141	10.9	1.00	--
Somatic	2	0.53	3.18	0.11
Germline	8	3.7	2.10	0.08
Any type of variations	10	3.7	2.29	0.02
489				
Wild	65	10.5	1.00	--
Somatic	11	20.1	0.55	0.20
Germline	75	9.4	1.13	0.54
Any type of variations	86	11.2	1.03	0.85
513				
Wild	145	10.5	1.00	--
Somatic	1	--	1.25	0.99
Germline	5	9.2	1.61	0.29
Any type of variations	6	9.8	1.18	0.71
[*]d514				
Wild	123	9.8	1.00	--
Somatic	9	12.4	0.76	0.52
Germline	19	12.9	0.77	0.41
Any type of variations	28	12.9	0.76	0.32
[*]d517				
Wild	146	10.3	1.00	--
Somatic	--	--	--	--
Germline	5	10.9	0.97	0.96
Any type of variations	5	10.9	0.97	0.96
[**]i302				
Wild	93	10.9	1.00	--
Somatic	12	10.2	1.12	0.76
Germline	46	9.8	0.94	0.79
Any type of variations	58	10.2	0.98	0.91
[**]i309				
Wild	136	10.3	1.00	--
Somatic	7	12.1	0.61	0.39
Germline	8	10.9	0.94	0.88
Any type of variations	15	12.1	0.79	0.51

N, Number of patients. [*]Deletion [**]Insertion.

Supplementary Table 3: Restricted mean survival time of event free survival for mitochondrial D-loop variations

Positions and type of variations	*N*	Survival (months) Mean ± SE	Difference (95% CI)	*P*
16184				
Wild	144	13.1 ± 0.8	--	--
Somatic	1	24.0 ± 0.0	10.9 (9.5 to 12.4)	≤0.01
Germline	6	13.4 ± 3.9	0.3 (-7.5 to 8.1)	0.93
Any type of variations	7	14.9 ± 3.5	1.9 (-5.2 to 8.9)	0.60
16318				
Wild	141	12.9 ± 0.8	--	--
Somatic	1	--	--	--
Germline	9	17.1 ± 2.3	4.1 (-0.6 to 8.9)	0.09
Any type of variations	10	16.2 ± 2.3	3.2 (-1.5 to 7.9)	0.18
198				
Wild	144	13.1 ± 0.8	--	--
Somatic	2	24.0 ± 0.0	10.9 (9.4 to 12.4)	≤0.01
Germline	5	--	--	--
Any type of variations	7	14.6 ± 3.1	1.6 (-4.6 to 7.8)	0.61

Abbreviations: CI, Confidence interval; N, Number of patients; SE, Standard error.

Supplementary Table 4: Overall survival for mitochondrial D-loop variations (Cox proportional)

Positions and type of variations	*N*	Median Survival Months	Hazard ratio	*P*
16051				
Wild	140	20.1	1.00	--
Somatic	1	--	5.21	0.10
Germline	10	12.5	0.89	0.80
Any type of variations	11	12.5	1.03	0.94
16111				
Wild	144	20.3	1.00	--
Somatic	2	0.3	28.9	≤0.01
Germline	5	13.3	2.2	0.08
Any variations	7	9.8	3.0	≤0.01
16126				
Wild	135	21.0	1.00	--
Somatic	2	0.5	7.41	≤0.01
Germline	14	9.2	2.08	0.03
Any variations	16	9.1	2.32	≤0.01
16129				
Wild	131	16.2	1.00	--
Somatic	3	8.2	0.53	0.53
Germline	17	16.2	0.79	0.51
Any variations	20	16.9	0.75	0.41
16172				
Wild	142	10.9	--	--
Somatic	3	6.8	0.61	0.62
Germline	6	6.3	2.02	0.12
Any variations	9	6.8	1.46	0.36
16189				
Wild	142	17.0	1.00	--
Somatic	3	0.56	1.82	0.40
Germline	6	8.63	0.54	0.39
Any variations	9	8.93	0.84	0.73
16223				
Wild	14	20.4	1.00	--
Somatic	11	8.2	0.95	0.91
Germline	96	15.4	1.12	0.65
Any variations	107	16.2	1.10	0.69
16278				
Wild	138	10.5	--	--
Somatic	2	1.1	6.37	0.01
Germline	11	13.0	0.58	0.23
Any variations	13	6.8	0.78	0.54
16292				
Wild	145	16.9	1.00	--
Somatic	1	--	1.23	0.99
Germline	5	13.3	0.66	0.55
Any variations	6	16.9	0.54	0.39
16294				
Wild	145	18.9	1.00	--
Somatic	--	--	--	--
Germline	6	10.9	0.82	0.74
Any variations	6	10.9	0.82	0.74
16311				
Wild	120	20.1	1.00	--
Somatic	9	6.3	1.84	0.12
Germline	22	16.2	1.05	0.86
Any variations	31	14.2	1.24	0.40
16318				
Wild	141	15.4	1.00	--
Somatic	1	--	4.22	0.99
Germline	9	--	0.28	0.08
Any variations	10	--	0.25	0.05
16362				
Wild	129	18.9	1.00	--
Somatic	4	9.7	2.45	0.08
Germline	18	12.1	0.67	0.32
Any variations	22	12.7	0.91	0.78
16390				
Wild	145	10.9	1.00	--
Somatic	1	--	6.16	0.07
Germline	5	18.0	1.72	0.23
Any variations	6	9.3	1.95	0.11

16519				
Wild	27	16.2	1.00	--
Somatic	5	20.3	0.63	0.54
Germline	119	16.9	1.00	0.99
Any variations	124	18.9	0.98	0.95
61				
Wild	140	16.9	1.00	--
Somatic	11	20.7	0.78	0.59
Germline	0	--	--	--
Any variations	11	20.7	0.78	0.59
73				
Wild	6	4.4	1.00	--
Somatic	6	14.2	0.59	0.43
Germline	139	20.1	0.44	0.04
Any variations	145	20.1	0.45	0.08
146				
Wild	132	20.4	1.00	--
Somatic	6	7.9	1.71	0.29
Germline	13	10.0	1.95	0.04
Any variations	19	8.9	1.87	0.03
151				
Wild	142	18.9	1.00	--
Somatic	1	--	14.4	0.01
Germline	8	10.9	0.87	0.80
Any variations	9	10.5	1.07	0.86
152				
Wild	101	20.3	1.00	--
Somatic	7	4.8	1.99	0.14
Germline	43	18.1	1.01	0.95
Any variations	50	12.5	1.11	0.64
195				
Wild	106	18.9	1.00	--
Somatic	9	10.3	1.10	0.83
Germline	36	20.1	0.97	0.92
Any variations	45	15.1	0.99	0.99
199				
Wild	145	20.1	1.00	--
Somatic	1	--	4.62	0.99
Germline	5	3.8	1.82	0.24
Any variations	6	9.3	1.34	0.57
204				
Wild	135	16.9	1.00	--
Somatic	2	0.53	31.5	≤0.01
Germline	14	20.3	0.88	0.74
Any variations	16	20.3	1.12	0.74
207				
Wild	142	20.1	1.00	--
Somatic	4	3.7	1.06	0.93
Germline	5	9.5	2.05	0.12
Any variations	9	9.5	1.61	0.22
263				
Wild	3	--	1.00	--
Somatic	9	16.9	0.23	0.26
Germline	139	20.1	0.34	0.27
Any variations	148	20.1	0.34	0.36
482				
Wild	141	20.3	1.00	--
Somatic	2	0.53	3.67	0.07
Germline	8	9.1	2.01	0.10
Any variations	10	9.1	2.26	0.02
489				
Wild	65	18.9	1.00	--
Somatic	11	20.4	0.77	0.59
Germline	75	13.8	1.19	0.44
Any variations	86	20.1	1.12	0.59
513				
Wild	145	20.1	1.00	--
Somatic	1	--	4.63	0.99
Germline	5	9.5	1.65	0.32
Any variations	6	9.8	1.23	0.67

Supplementary Table 5: Restricted mean survival time of overall survival for mitochondrial D-loop variations

Positions and type of variations	*N*	Survival (months) Mean ± SE	Difference (95% CI)	*P*
16093				
Wild	143	12.6 ± 0.5	--	--
Somatic	2	11.1 ± 0.0	-1.5 (-2.6 to -0.4)	≤0.01
Germline	6	9.9 ± 3.6	-2.7 (-9.8 to 4.5)	0.46
Any variations	8	10.2 ± 2.9	-2.4 (-8.2 to 3.4)	0.42
16184				
Wild	144	15.2 ± 0.8	--	--
Somatic	1	24.0 ± 0.0	8.8 (7.3 to 10.3)	≤0.01
Germline	6	16.3 ± 3.3	1.1 (-5.5 to 7.6)	0.74
Any variations	7	17.5 ± 2.9	2.3 (-3.6 to 8.2)	0.45
16192				
Wild	144	15.4 ± 0.8	--	--
Somatic	2	12.4 ± 0.0	-3.1 (-4.6 to -1.6)	≤0.01
Germline	5	13.8 ± 4.2	-1.7 (-10.0 to6.7)	0.69
Any variations	7	13.4 ± 3.9	-2.1 (-9.9 to 5.8)	0.61
16209				
Wild	143	15.3 ± 0.8	--	--
Somatic	2	12.4 ± 0.0	-3.1 (-4.6 to -1.6)	0.03
Germline	6	19.5 ± 2.8	4.2 (-1.5 to 9.8)	0.14
Any variations	8	15.2 ± 3.7	-0.1 (-7.5 to 7.3)	0.98
16304				
Wild	144	15.2 ± 0.8	--	--
Somatic	1	12.4 ± 0.0	-2.8 (-4.6 to -1.6)	0.12
Germline	6	19.8 ± 2.9	4.6 (-1.4 to 10.5)	0.13
Any variations	7	17.1 ± 3.9	1.8 (-6.0 to 9.7)	0.65
16327				
Wild	144	15.5 ± 0.7	--	--
Somatic	2	13.5 ± 0.0	-2.0 (-3.5 to -0.5)	≤0.01
Germline	5	10.9 ± 4.9	-4.6 (-14.5 to5.3)	0.20
Any variations	7	11.6 ± 4.1	-3.9 (-12.1 to4.3)	0.35
150				
Wild	130	15.1 ± 0.8	--	--
Somatic	5	19.2 ± 2.1	4.1 (-0.3 to 8.5)	0.06
Germline	16	16.2 ± 2.0	1.1 (-3.2 to 5.4)	0.60
Any variations	21	16.8 ± 1.7	1.8 (-1.9 to 5.6)	0.34
198				
Wild	144	15.2 ± 0.8	--	--
Somatic	2	24.0 ± 0.0	8.7 (7.2 to 10.3)	≤0.01
Germline	5	--	--	--
Any variations	7	17.1 ± 2.9	1.9 (-3.9 to 7.7)	0.52

Abbreviations: CI, Confidence interval; N, Number of patients; SE, Standard error.

Supplementary Table 6: Event free survival and overall survival for any type of nucleotide change in mitochondrial DNA D-loop variations

Parameters	*N*	Overall survival		Event free survival	
		HR (95% CI)	*P*	HR (95% CI)	*P*
**Somatic variations**					
Change into A	119	1.00	0.67	1.00	0.82
	32	0.9 (0.5–1.5)		0.9 (0.6–1.5)	
Change into G	126	1.00	0.57	1.00	0.66
	25	0.8 (0.4–1.5)		1.1 (0.6–1.8)	
Change into C	96	1.00	0.96	1.00	0.68
	55	0.9 (0.6–1.5)		1.1 (0.7–1.6)	
Change into T	87	1.00	0.02	1.00	0.07
	64	1.6 (1.0–2.5)		1.4 (0.9–2.1)	
**Germline variations**					
Change into T	6	1.00	0.52	1.00	0.68
	145	1.6 (0.4–6.3)		1.2 (0.4–4.0)	
Change into G	97	1.00	0.59	1.0	0.88
	54	1.1 (0.7–1.7)		1.0 (0.6–1.5)	
Change into C	27	1.00	0.57	1.00	0.69
	124	1.2 (0.6–2.1)		1.1 (0.6–1.8)	
**Any type of variations change**					
Total change into A					
≤2 Positions	81	1.00	0.89	1.00	0.70
≥2 Positions	70	0.9 (0.6–1.5)		0.9 (0.7–0.6)	
Total change into G					
0 Positions	79	1.00		1.00	1.6
≥0 Positions	72	1.1 (0.7–1.6)	0.67	1.1 (0.7–1.6)	
Total change into C					
≤2 Positions	98	1.00		1.00	0.81
≥2 Positions	53	1.2 (0.8–1.8)	0.43	1.0 (0.7–1.6)	
Total change into T					
≤2 Positions	37	1.00		1.00	0.31
≥2 Positions	114	2.0 (1.1–3.6)	0.02	1.3 (0.8–2.0)	
